# Simulation and Experimental Study of Multi-Grain Diamond Cutting of Monocrystalline Silicon

**DOI:** 10.3390/mi17020186

**Published:** 2026-01-29

**Authors:** Guofu Luo, Shuo Sun, Liwei Li, Yan Lv, Wuyi Ming

**Affiliations:** 1 Henan Provincial Key Laboratory of Intelligent Manufacturing of High-End Equipment, Zhengzhou University of Light Industry, Zhengzhou 450002, China; luoguofu@zzuli.edu.cn (G.L.); sunshuo202304@163.com (S.S.); 2 Faculty of Engineering, Huanghe University of Science and Technology, Zhengzhou 450061, China; 3 Department of Optical, Mechanical and Electrical, Zhejiang A&F University, Hangzhou 311300, China; yan.lv@outlook.com; 4 Guangdong HUST Industrial Technology Research Institute, Huazhong University of Science and Technology, Dongguan 523808, China

**Keywords:** diamond grits, cutting force, monocrystalline silicon, surface roughness, surface morphology

## Abstract

Diamond wire sawing, as the core process for monocrystalline silicon wafering, has gained widespread application in the photovoltaic and microelectronics industries due to its high efficiency and low material loss. This study investigates the cutting mechanism of monocrystalline silicon with (100) crystal orientation under multi-abrasive and multi-scratch conditions using explicit finite element dynamics simulation. It focuses on analyzing the effects of radial spacing and height difference between abrasive grains on surface morphology, cutting force, and residual stress. Based on the Johnson-Holmquist-II (JH-II) constitutive model, a high-precision three-dimensional finite element simulation model was constructed. Simulation results indicate that the spacing and height difference between abrasive grains significantly affect the grain-to-grain coupling, thereby influencing the peak cutting force and the surface damage characteristics of the scratches. To address cutting force and residual stress responses, this study proposes an algorithmic optimization scheme based on a multifactor orthogonal experimental design. The analysis indicates that the optimal parameters—U = 1385 m/min, V = 142°, and W = 6.2 μm—reduce residual stress by 33% and cutting force by 75%.

## 1. Introduction

As the global energy structure accelerates its transition toward low-carbon and renewable sources, photovoltaic power generation has emerged as a core force in future energy development [1]. Monocrystalline silicon wafers, serving as the fundamental substrate for photovoltaic cells and semiconductor devices, directly determine device efficiency and lifespan through their manufacturing quality [2,3]. Monocrystalline silicon has become the dominant material with over 90% market share due to its high electron mobility, excellent thermal conductivity, and stable chemical and mechanical properties [4]. However, its high hardness, brittleness, and pronounced crystal anisotropy make it highly susceptible to microcracks, lattice defects, and subsurface damage during cutting and ultra-precision machining. This not only reduces yield rates but also significantly increases the thickness and cost of subsequent polishing to remove damaged layers [5]. Therefore, exploring advanced cutting technologies that ensure high cutting efficiency while significantly suppressing surface and subsurface damage has become a critical task for driving high-quality development in the photovoltaic and semiconductor industries.

**Figure 1:**
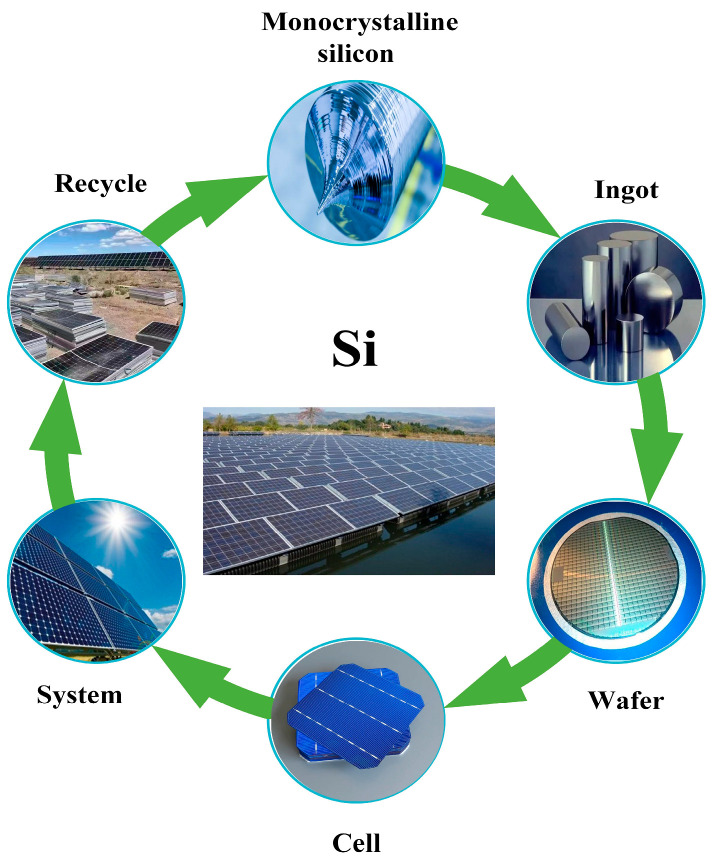
A diagram of the whole supply chain of photovoltaic manufacturing.

The primary processing methods for monocrystalline silicon include traditional and ultra-precision techniques such as mechanical polishing, lapping, single-point diamond turning, and micro-grinding [6,7,8]. All these methods require the use of diamond micro-powder or cutting tools to meet material removal demands under its highly hard and brittle characteristics [9]. However, these methods exhibit low processing efficiency on hard and brittle materials, significant material loss, and a tendency to cause substantial surface and subsurface damage. Consequently, diamond wire saw cutting remains the mainstream industrial approach [10]. Diamond wire saws (DWS) have comprehensively replaced traditional free-abrasive wire saw technology due to their advantages of high cutting efficiency (wire speeds up to 3000 m/min), superior surface quality, and environmental friendliness, becoming the core process for photovoltaic cell and semiconductor wafer fabrication [11,12]. Figure 1 illustrates the schematic of the entire photovoltaic cell manufacturing supply chain. Monocrystalline silicon holds a pivotal position in the global photovoltaic industry due to its mature material preparation technology and outstanding photoelectric conversion efficiency [13]. However, the high hardness and dynamic impact characteristics of diamond abrasives in the DWS process lead to more complex surface/subsurface damage in silicon wafers. Furthermore, the coupling between wire saw parameters and silicon crystal anisotropy significantly influences crack propagation paths and damage distribution [14]. There is an urgent need to elucidate the dynamic relationship between material removal and crack evolution at the mechanistic level to resolve the process contradiction between “high cutting efficiency and low surface damage.”

Researchers characterized surface topography and damage features such as periodic concave-convex structures, microcrack distribution, and frozen dislocations in wire saw cross-sections, providing key strategies for reducing machining damage through parameter co-design [15]. Additionally, experimental studies on the effects of wire saw wear on cutting forces and wafer surface quality revealed correlations between wear levels and machining performance, offering references for optimizing wire saw lifespan and cost [16]. Wan et al. [17] derived expressions for stress distributions around indenters during silicon single-crystal scribing based on classical Boussinesq and Cerruti theories. Fang [18] investigated grain dislocations in silicon crystals during nanoindentation and scribing using stress distribution analysis, demonstrating phase transitions within crystals as load increases. These two papers theoretically elucidate the stress field and dislocation behavior of abrasive particles during silicon wafer scribing. Chen et al. [19] employed molecular dynamics simulations to investigate surface morphology changes on different crystal planes during silicon wafer grinding, explaining the principle of sawing force variation from the perspective of grain boundary dislocations. Cheng et al. [20] conducted experiments on micro-grinding monocrystalline silicon across two distinct ranges of cutting depth and feed rate. They established the relationship between grinding parameters and monocrystalline silicon crack length, as well as the minimum undeformed chip thickness required for ductile removal. Academic research on this process primarily focuses on cutting mechanism modeling and numerical simulation. Mathematical analytical models, molecular dynamics, and finite element methods are employed to predict cutting forces, surface roughness, and subsurface damage, thereby guiding parameter optimization. Cutting mechanism modeling is crucial to this research.

Scratch testing is a crucial method for investigating the removal mechanism of hard, brittle materials. In recent years, single-grain experiments and dual-scratch tests have been widely applied to analyze the abrasion mechanism of brittle materials [16]. Ge et al. [21] conducted variable-load scratch tests on single-crystal silicon (100) orientation using a Berkovich indenter, determining a critical load of 26 mN where the material removal mode transitions from ductile to brittle (DBT). Wang et al. [22] employed a conical indenter to scratch along the (110) grain orientation of single-crystal silicon, determining a residual critical depth of 0.162 μm for the DBT using a three-dimensional surface optical profiler. Furthermore, Wang et al. [23] established a stress field model for dual scratching on single-crystal silicon carbide using the superposition principle and analyzed the interaction of transverse cracks during the dual-scratch process. Current research primarily focuses on single-abrasive-particle single-scratch studies, with material investigations concentrated on silicon carbide, ceramics, and similar materials. Research on multi-abrasive-particle multi-scratch studies involving single-crystal silicon materials remains limited.

Although finite element simulations and experimental studies have made significant progress in elucidating the cutting mechanisms of diamond wire saws on monocrystalline silicon, several key scientific issues remain inadequately addressed. Existing research predominantly focuses on single abrasive particles and single scratches, with material investigations concentrated on silicon carbide, ceramics, and similar materials. Research on the interaction of single-crystal silicon under multi-abrasive-particle and multi-scratch conditions remains scarce. The coupled effects of radial spacing and height difference between abrasive particles on stress field evolution and material removal patterns have not been fully characterized. The relationship between residual stress and cutting force under varying process parameters remains unclear. Existing optimization schemes are predominantly based on single- or dual-factor analysis, lacking comprehensive multi-objective trade-off strategies that balance cutting efficiency, surface quality, and tool life. To address these challenges, this study establishes an integrated research framework combining explicit dynamic finite element simulation, multi-parameter orthogonal experimental design, and multi-objective genetic algorithm optimization. A three-dimensional finite element model incorporating the Johnson-Holmquist-II (JH-II) constitutive model reveals the dynamic cutting mechanism under multi-abrasive-multi-scratch conditions, with a focus on decoupling the effects of radial spacing and axial height difference. Orthogonal design established the hierarchy of primary controlling factors and their nonlinear interactions. The multi-objective genetic algorithm obtained Pareto optimal solutions for the residual stress-cutting force trade-off, providing guidance for abrasive arrangement optimization and process parameter coordination. This approach supports theoretical understanding of multi-abrasive synergistic cutting mechanisms and engineering practices for high-quality monocrystalline silicon wafer fabrication.

## 2. Model Establishment

### 2.1. Establishment of a Constitutive Model for Monocrystalline Silicon

Currently, the primary constitutive models for simulating brittle-hard materials such as monocrystalline silicon include the Johnson-Holmquist-Beissel, Johnson-Holmquist-II, and Drucker-Prager models [1]. The JH-II constitutive model accurately simulates the mechanical response and failure behavior of silicon carbide under large strains and high strain rates [24], making it the optimal choice for finite element simulations in this study [25]. The JH-II model consists of three components: strength, damage, and pressure. The material strength can be expressed by the following normal equivalent stress:

(1) $$\sigma^{*}=\sigma_{i}^{*}-D\left(\sigma_{i}^{*}-\sigma_{f}^{*}\right)$$

Among these, $\sigma_{i}^{*}$ denotes the normalized complete equivalent stress, $\sigma_{f}^{*}$ represents the normalized fracture equivalent stress, and D is the damage variable [26]. The normalized equivalent stresses ($\sigma^{*}$, $\sigma_{i}^{*}$, and $\sigma_{f}^{*}$) share the general form $\sigma^{*}=\frac{\sigma }{\sigma_{HEL}}$, where σ is the actual von Mises equivalent stress and $\sigma_{HEL}$ is the equivalent stress at the Hugoniot elastic limit. The normalized intact and fracture equivalent stresses can be expressed as functions of pressure and strain rate:

(2) $$\sigma_{i}^{*}=A(P^{*}+T^{*}{)}^{N}(1+Cln\;{\dot{\epsilon }}^{*})\leq \sigma_{i}^{max}$$

(3) $$\sigma_{f}^{*}=B(P^{*}{)}^{M}(1+Cln\;{\dot{\epsilon }}^{*})\leq \sigma_{f}^{max}$$

(4) $$P^{*}=\frac{P}{P_{HEL}}$$

(5) $$T^{*}=\frac{T}{P_{HEL}}$$

In the equation, *A* is the intact normalized strength coefficient, *B* is the fractured normalized strength coefficient, *C* is the strain rate-dependent strength coefficient, *M* is the fracture strength index, and N is the intact strength index. $\sigma_{i}^{max}$ and $\sigma_{f}^{max}$ are the normalized intact equivalent stress maximum and the normalized fractured equivalent stress maximum, respectively [27]. $P^{*}$ denotes the normalized pressure, $T^{*}$ denotes the normalized maximum hydrostatic pressure, P denotes the actual pressure, $P_{HEL}$ denotes the pressure at the Hugoniot elastic limit, and T denotes the maximum hydrostatic pressure the material can withstand [28].

The damage evolution criterion is analogous to the Johnson–Cook model, where the damage parameter ω can be determined by the following equation:

(6) $$\omega =\sum \frac{\Delta {\bar{\epsilon }}^{pl}}{{\bar{\epsilon }}_{f}^{pl}\left(P\right)}$$

(7) $${\bar{\epsilon }}_{f}^{pl}=D_{1}(P^{*}+T^{*}{)}^{D_{2}},{\;\bar{\epsilon }}_{f,min}^{pl}\leq {\bar{\epsilon }}_{f}^{pl}\leq {\bar{\epsilon }}_{f,max}^{pl}$$

The relationship between pressure and density is expressed as follows:

(8) $$P=\left\{\begin{array}{cc} K_{1}\mu +K_{2}\mu^{2}+K_{3}\mu^{3} & \mu ⩾0\\ K_{1}\mu & \mu <0 \end{array}\right.$$

(9) $$\mu =\frac{\rho }{\rho_{0}}-1$$

In the equation, K_1_, K_2_, and K_3_ represent the bulk modulus, second pressure coefficient, and third pressure coefficient, respectively; ρ denotes the current density; and ρ_0_ is the reference density.

### 2.2. Simulation Models

This study employs the ABAQUS/Explicit dynamics algorithm to simulate the material removal mechanism during the multi-abrasive, multi-scratch machining of single-crystal silicon using diamond tools. Considering the nonlinear dynamic response characteristics of hard brittle materials under high-strain-rate impact loading, the explicit integration algorithm effectively handles complex physical phenomena such as material failure, contact nonlinearity, and large geometric deformation. This provides a reliable numerical computational foundation for revealing the dynamic interaction mechanism between diamond abrasive particles and the monocrystalline silicon substrate. Compared to traditional static analysis, this method is better suited for addressing transient dynamic issues in high-speed cutting processes, accurately capturing stress wave propagation and damage evolution during material removal.

To enhance computational efficiency and focus on core physical mechanisms, this model emphasizes primary cutting dynamics while temporarily treating nonlinear complexities—such as thermo-mechanical-electrical coupling effects, acoustic emission phenomena, and multiscale damage evolution—as higher-order variables [25]. Based on typical diamond wire saw cutting conditions, modeling adheres to the following core boundary conditions:

(1) Monocrystalline silicon material properties are set as an anisotropic elastoplastic medium, incorporating an orientation-dependent elastic modulus tensor;
(2) Diamond abrasive particles are defined as rigid bodies with constant linear velocity motion characteristics;
(3) The cutting interface employs a master-slave contact algorithm based on a penalty function, with a friction coefficient set to 0.15;
(4) Workpiece boundary conditions are set as fully constrained on the bottom surface and non-reflective on the side surfaces.

This model employs the governing equations of continuum mechanics, solving the momentum balance equation via an explicit central difference scheme. Combined with damage evolution parameters from the modified Johnson-Holmquist-II constitutive model and contact boundary conditions, it enables quantitative analysis of the dynamic removal process in mono-crystalline silicon materials. The monocrystalline silicon workpiece is discretized using C3D8R linear reduced-integration hexahedral elements, with a minimum mesh size set to accurately capture microcrack initiation and propagation behavior. The proposed model makes key assumptions based on actual industrial wire sawing conditions, providing a controllable numerical experimental platform for revealing micro-scale multi-abrasive synergistic cutting mechanisms and surface morphology evolution patterns, while balancing computational accuracy and solution efficiency.

### 2.3. Geometrical Modeling and Meshing

**Figure 2:**
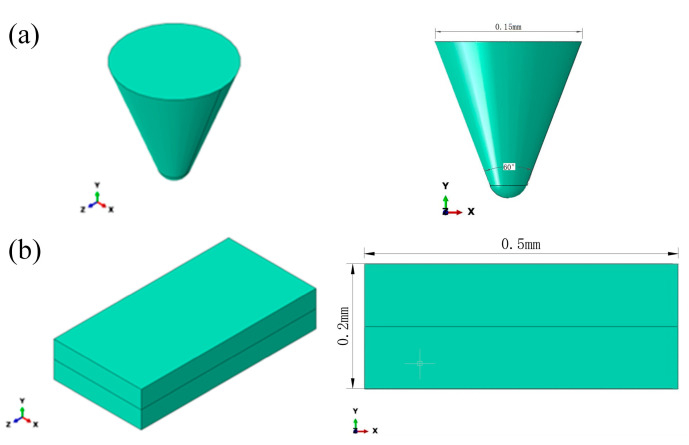
Schematic diagram of workpiece and tool dimensions. (**a**) Abrasive particle size; (**b**) Dimensions of Monocrystalline Silicon Workpieces.

In this study, geometric modeling and mesh generation are critical steps in the finite element simulation process, directly impacting the accuracy of simulation results and computational efficiency [29]. First, three-dimensional modeling was performed using SolidWorks 2024 to construct the cutting process geometry model of diamond abrasive particles interacting with a single-crystal silicon workpiece. This model encompasses the geometric shape of the diamond particles, the geometric features of the cutting zone, and the motion trajectory of the particles during the cutting process. The single-crystal silicon workpiece dimensions were 1 × 0.5 × 0.2 mm. To simulate the interaction between the diamond abrasive and the silicon substrate, the abrasive was modeled as a conical body with a 60° taper angle, a height of 0.16 mm, and a grain diameter of 0.15 mm, as shown in Figure 2.

**Figure 3:**
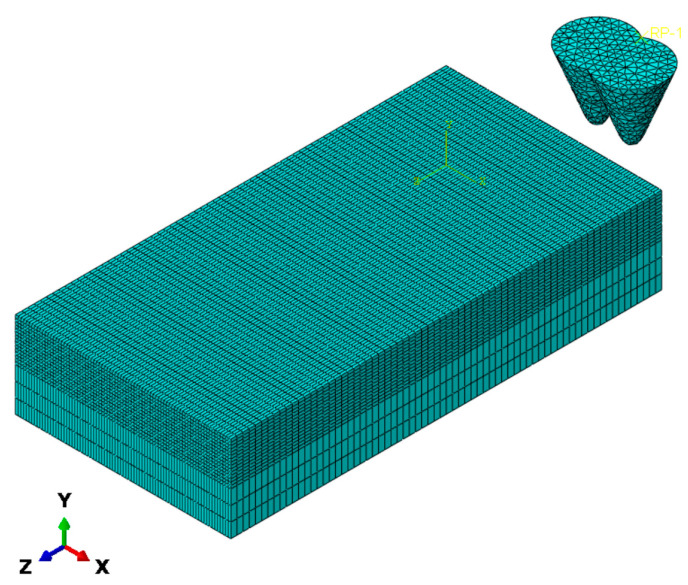
Schematic diagram of finite element mesh partitioning for abrasive particles and monocrystalline silicon.

For mesh generation, ABAQUS 2024 was employed for both meshing and post-processing. Considering the complex stress state and material failure behavior in the cutting region, adaptive mesh refinement technology was adopted in this study. Local meshing refinement was applied in the cutting contact area, with the minimum element size set to 0.005 mm to ensure accurate capture of microcrack initiation and propagation processes. A gradient mesh transition was applied to the far-field region to balance computational accuracy and efficiency, preventing computational resource wastage. The workpiece was entirely discretized using C3D8R linear-reduced-integration hexahedral elements, a mesh type that enhances computational accuracy and more precisely simulates material removal processes. Diamond abrasive grains were modeled as rigid bodies and discretized using C3D4 tetrahedral elements. The grid division is shown in Figure 3.

During the mesh generation process, mesh quality checks and mesh independence checks were implemented to ensure the mesh quality meets computational requirements. The mesh independence check guarantees that computational results stabilize as mesh density increases, preventing significant errors arising from variations in mesh refinement. The mesh quality check ensures the quality of the mesh within the computational domain, particularly in contact regions, which is crucial for enhancing computational accuracy and convergence. Numerical solution employed the explicit central difference method within ABAQUS’s explicit dynamics module, with time steps automatically determined based on Courant-Friedrichs-Lewy stability criteria. Typical time steps were approximately 10^−9^ s. To guarantee convergence and stability of numerical solutions, the total computation time per simulation was set to 2 ms, sufficient to encompass the entire multi-abrasive particle scratching process.

### 2.4. Model Parameter Setting

In finite element simulations, the accurate setting of model parameters is crucial for the credibility and precision of results. In this study, the physical property parameters of monocrystalline silicon and diamond were selected based on existing literature and experimental results of material characteristics. To ensure computational consistency and reliability, the T-mm-s unit system was adopted, with the physical properties of diamond and monocrystalline silicon shown in Table 1 [30]. Key parameters for monocrystalline silicon, including density, Young’s modulus, and Poisson’s ratio, were precisely defined according to experimental data to accurately reflect its mechanical behavior during the machining of high-hardness brittle materials.

For the constitutive behavior of monocrystalline silicon materials, this study employs the JH-II constitutive model, which effectively describes the nonlinear dynamic response and failure behavior of materials under high-speed, high-strain-rate conditions. The JH-II model incorporates three sets of parameters: strength, damage, and pressure. It simulates crack propagation, plastic deformation, and material removal during diamond abrasive scribing. Specific model parameters are listed in Table 2 [31]. These parameters were selected based on experimentally determined values for single-crystal silicon from the literature, combined with the material’s stress–strain curve and failure criteria. This ensures the simulation model accurately reflects the material’s true cutting characteristics.

During the simulation process, all physical parameter settings strictly adhere to the ABAQUS platform simulation requirements. Particularly in defining material properties, considering the anisotropic behavior of monocrystalline silicon, where elastic modulus and strength exhibit significant variations across different crystal orientations, an orientation-dependent elastic modulus tensor was incorporated into the model to further enhance simulation accuracy. Furthermore, by meticulously adjusting the damage and stress parameters within the JH-II model, the simulation ensures that the material failure mode aligns with the crack propagation behavior observed in actual cutting processes. These precise model parameter settings provide a robust theoretical foundation for subsequent predictions of cutting forces and surface roughness.

## 3. Simulation Results and Analysis

**Figure 4:**
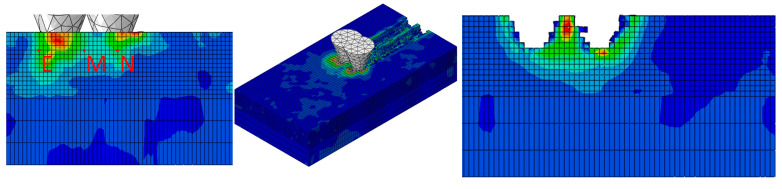
Simulation flowchart for diamond abrasive cutting of monocrystalline silicon.

To visually reveal the dynamic mechanical response process of diamond abrasive cutting on monocrystalline silicon, this chapter conducted systematic finite element simulations using the ABAQUS/Explicit dynamics module. Figure 4 illustrates the typical evolution of the von Mises stress field during multi-abrasive cutting, showing from left to right: the initial cutting stage, the steady-state cutting stage, and the completed cutting stage. The stress contour clearly indicates that during the initial cutting phase, stresses are concentrated at the abrasive-workpiece interface. As cutting progresses, the stress field propagates deeper into the workpiece, forming distinct high-stress zones. After cutting completion, significant residual stress gradients persist within the workpiece, exhibiting non-uniform distribution characteristics within the scratched region.

The principal stress concentration point N is positioned 0.015 mm below the center of the primary particle scratch, reflecting peak stress response under single-particle action. Point M, the center of the particle coupling zone, is positioned at the geometric midpoint between adjacent particle scratches and shares the same depth as point N. Its stress value exhibits significant variation with particle spacing. Point E, the boundary damage propagation point, is located 0.15 mm outside the scratch edge at the same depth as point N. It characterizes lateral crack initiation tendencies and surface damage characteristics. An RP reference point is established at the bottom of the right abrasive to extract cutting force data. These three points form a complete mechanical response chain: “primary cutting zone → coupled influence zone → boundary damage zone.” By extracting von Mises stress and cutting force data at each point during the steady-state cutting phase, the influence of abrasive layout parameters on stress distribution and material removal mechanisms can be revealed. This provides a theoretical basis for optimizing grinding process parameters and predicting machined surface quality.

### 3.1. Effect of Abrasive Radial Spacing on Surface Topography

#### 3.1.1. Analysis of Residual Stresses and Surface Topography at Different Spacing

**Figure 5:**
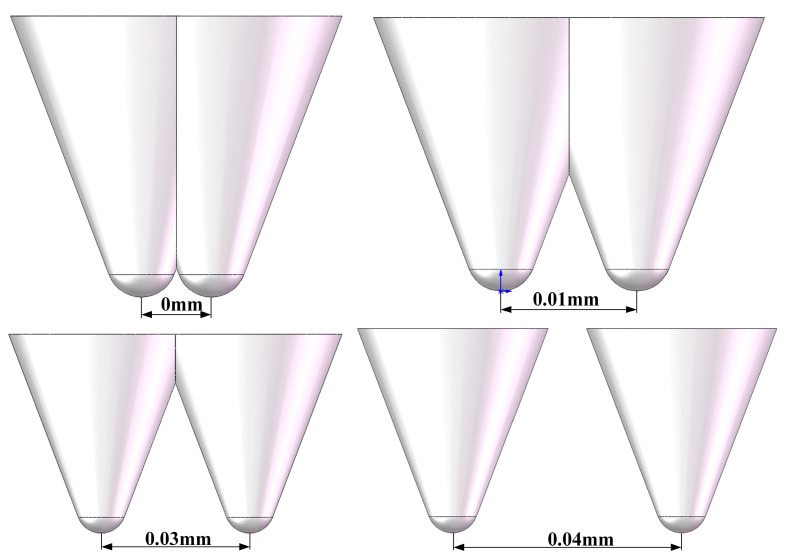
Schematic Diagram of Abrasive Grains with Different Radial Spacing.

**Figure 6:**
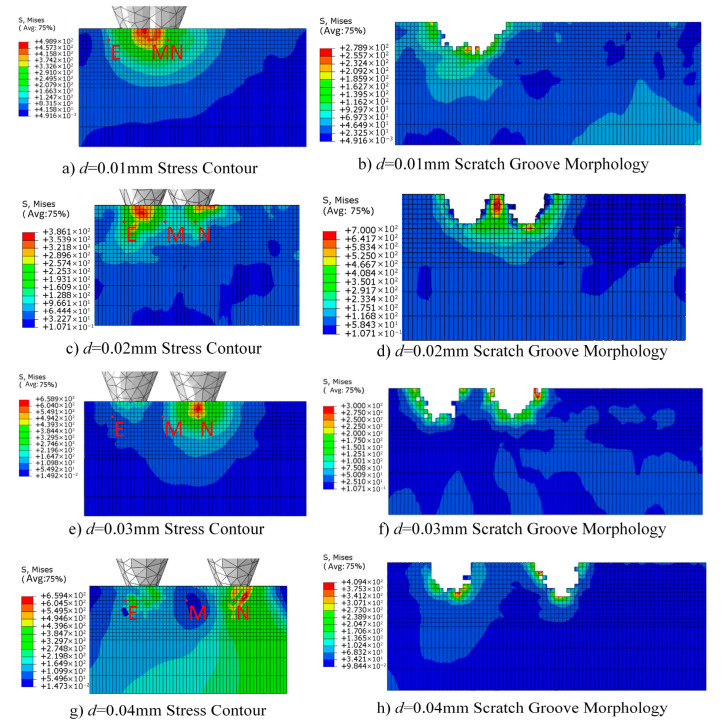
Stress contour maps and scratch morphology at different radial spacings.

Figure 5 shows the distribution of abrasive particles at different radial spacings. Figure 6 illustrates the influence of radial spacing on the surface morphology and residual stress distribution of monocrystalline silicon. When radial spacing d = 0.01 mm, the action zones of the two abrasive particles overlap significantly, resulting in a pronounced superposition of stress fields. The von Mises stress at point M reaches 460 MPa. At this point, the scratched surface exhibits typical brittle fracture characteristics. A continuous high-stress zone forms between abrasive particles, with material removal dominated by brittle fracture, leading to significant deterioration in surface roughness. Stress contour plots reveal a saddle-shaped distribution of high-stress zones (>400 MPa) between the two abrasive particles. This indicates that microcrack networks induced by the preceding particle provide preferential propagation paths for subsequent particles, leading to increased uncontrollability in material removal.

The aforementioned phenomenon can be attributed to the dynamic coupling effect between abrasive particles and the stress field superposition mechanism. When the radial spacing between abrasive particles is small, the stress fields of adjacent particles strongly superimpose, forming continuous high-stress regions within the workpiece. According to fracture mechanics theory, the stress intensity factor is proportional to the stress field intensity. When the superimposed stress exceeds the fracture toughness of monocrystalline silicon, microcracks propagate rapidly and interconnect, leading to large-scale material removal via a brittle fracture mode. Furthermore, the plastic deformation zones and damage regions caused by preceding abrasive particles have not fully recovered. Subsequent cutting actions by new particles effectively reload these “pre-damaged” areas, significantly reducing local strength and toughness while exacerbating surface fragmentation and subsurface damage.

According to indentation fracture mechanics theory, the material removal mode of hard brittle materials during machining is determined by the relative relationship between the abrasive cutting depth and the critical cutting depth [32]. When the actual cutting depth of a single abrasive grain is below the critical value, material removal primarily occurs through plastic deformation and shear flow, resulting in a ductile domain on the machined surface. When the cutting depth exceeds the critical value, a median crack and transverse crack network form within the abrasive grain’s influence zone. The propagation and interaction of these cracks lead to material removal via a brittle fracture mechanism. The evolution of scratch morphology in Figure 6 clearly reveals the transition process of material removal mechanisms. When d ≤ 0.02 mm, the scratch edges exhibit typical “serrated” chipping features, directly indicating lateral crack instability propagation. Conversely, when d ≥ 0.03 mm, the scratch edges become smooth and continuous, indicating that material removal is dominated by plastic flow, with crack initiation effectively suppressed. This transition corresponds to the critical brittle-to-ductile transition phenomenon in monocrystalline silicon processing. Its critical spacing is approximately 0.025–0.03 mm, correlating to 0.17–0.20 times the abrasive particle size. This provides a crucial reference for optimizing the particle arrangement in diamond wire saws.

**Figure 7:**
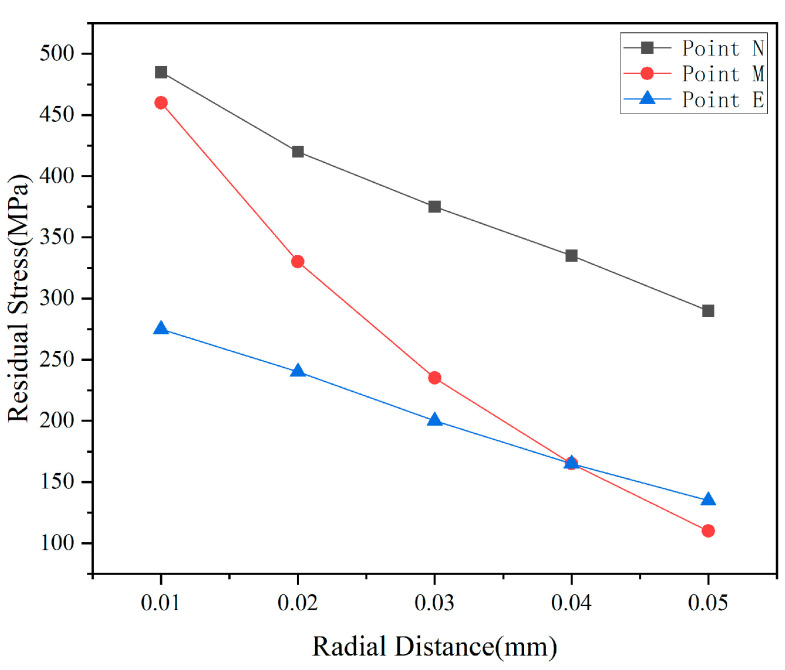
Residual stress at nodes as a function of radial spacing.

Figure 7 quantitatively illustrates the evolution of residual stresses at three monitoring points with radial spacing, revealing the spatial attenuation characteristics of stress field propagation. The stress in the primary cutting zone at point N monotonically decreased from 490 MPa (d = 0.01 mm) to 290 MPa (d = 0.05 mm), representing a 41% reduction. This indicates that even within the primary active zone, increasing the particle spacing can reduce peak stress by mitigating stress concentration. More notably, the stress at the coupling zone (Point M) exhibited a steep decline from 460 MPa to 110 MPa—a 76% reduction—clearly demonstrating the decisive influence of particle coupling effects on stress distribution. The stress variation in the boundary zone at point E was relatively gradual, decreasing from 280 MPa to 130 MPa. However, its absolute value remained significantly lower than that at points N and M, indicating rapid attenuation of stress waves during lateral propagation. This phenomenon is closely related to the anisotropic elastic properties of single-crystal silicon.

Notably, when the radial pitch exceeds 0.03 mm, the slopes of all three curves decrease significantly, indicating that the stress reduction rate tends to level off. This aligns with the influence radius of the abrasive stress field. Beyond this critical distance, increasing the spacing yields diminishing marginal benefits in residual stress reduction while reducing material removal rates. Therefore, considering both stress control and machining efficiency, a radial spacing of 0.025–0.035 mm is recommended. This range effectively suppresses abrasive coupling effects while ensuring adequate cutting density.

#### 3.1.2. Analysis of Cutting Forces at Different Spacing

**Figure 8:**
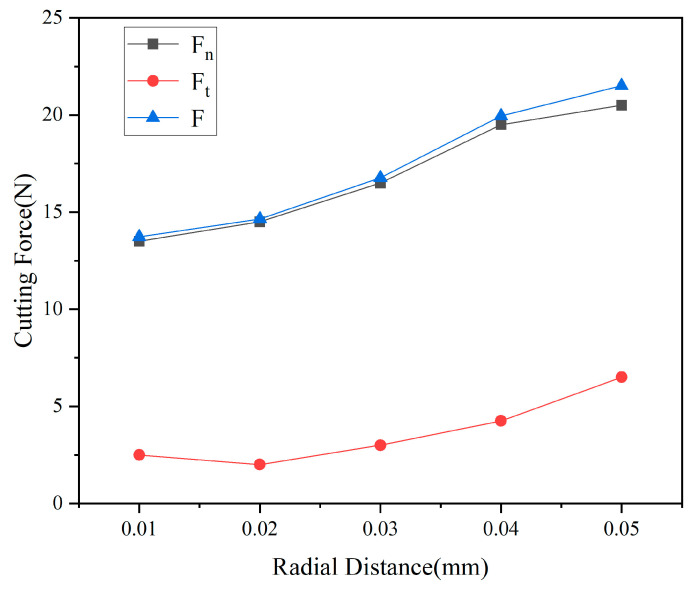
Effect of radial clearance on cutting force.

Figure 8 reveals the influence of radial spacing between abrasive particles on the normal force F_n_, tangential force F_t_, and resultant force F. When radial spacing d = 0.01 mm, the normal force F_n_ and resultant force F are 13.8 N and 13.9 N, respectively, while the tangential force Ft is only 2.4 N. At this extremely small particle spacing, subsequent abrasive particles almost entirely “tread” upon the plastic deformation zone formed by preceding particles. This reduces the effective cutting thickness of the material, resulting in relatively low tangential resistance. However, due to the intense superposition of stress fields, a high-density network of microcracks forms within the material. This significantly increases the normal compressive force required to overcome the material’s fracture resistance. Material removal at this stage primarily follows a “crushing-peeling” mechanism, with energy expenditure predominantly directed toward crack initiation and propagation rather than effective cutting deformation.

The underlying mechanism of this phenomenon can be explained from the perspective of micro-mechanics in abrasive-workpiece interactions. Under close spacing conditions, the residual stress field and damage zone induced by the preceding abrasive grain have not yet recovered, leading to a significant reduction in the material’s local yield strength and fracture toughness. Subsequent abrasive penetration acts as secondary loading on the “softened” zone, requiring lower normal forces. However, due to the material’s damaged state, cutting efficiency declines, manifested as a reduced proportion of tangential forces. Conversely, in wide-pitch configurations, abrasive particles act on “undisturbed” material surfaces, requiring higher normal forces for penetration and plastic deformation. However, material integrity is preserved, allowing plastic flow and shear to become the dominant material removal mechanisms. This increases the proportion of tangential forces and enhances cutting efficiency.

Comprehensive cutting force analysis yields the following process optimization recommendations: When minimizing surface damage is the objective, a radial clearance of d = 0.03–0.035 mm is optimal. At this setting, cutting forces are moderate, and the material removal mode approaches ductile-range processing, yielding the best surface integrity. When prioritizing material removal rate, the clearance can be appropriately reduced to 0.015–0.02 mm, though this entails some degradation in surface quality. For precision machining applications, a configuration with d ≥ 0.04 mm is recommended. Although the cutting force per abrasive grain is higher (F > 20 N), the material removal mechanism fully transitions into the plastic deformation regime, enabling the achievement of mirror-like surface roughness.

### 3.2. Effect of Abrasive Particle Height Difference on Simulation Results

#### 3.2.1. Surface Topography and Stress Contour Map

**Figure 9:**
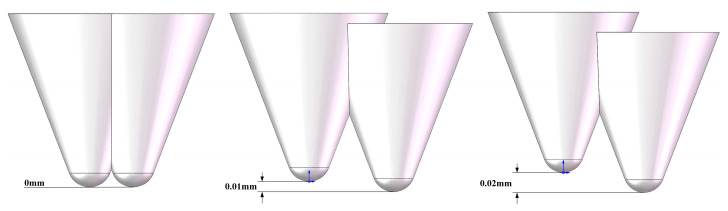
Schematic Diagram of Abrasive Particles at Different Height Differences.

During actual diamond wire saw cutting, axial height differences inevitably exist between abrasive grains due to uneven grain embedding positions, fluctuations in electroplating layer thickness, and inherent size variability of the grains themselves. These height differences significantly influence cutting behavior on monocrystalline silicon surfaces, directly affecting the consistency and predictability of machined surface quality. To systematically investigate the mechanism of this parameter, this study fixed the radial spacing between abrasive grains at 0.02 mm and examined the evolution of stress fields and surface topography characteristics as the height difference h varied within the range of 0–0.02 mm. Figure 9 illustrates schematic diagrams of abrasive particles at different height differences.

**Figure 10:**
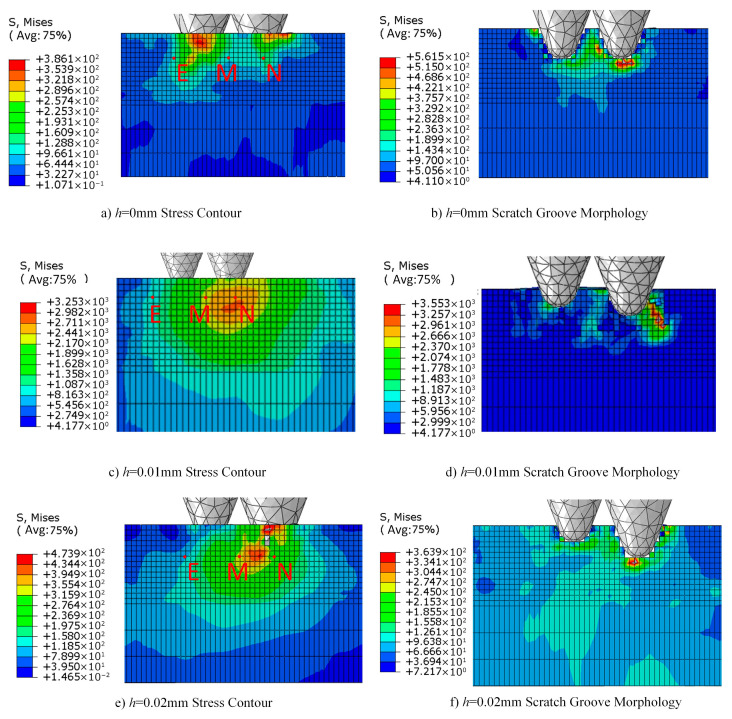
Stress contour maps and scratch morphology at different axial spacings.

Figure 10 displays stress contour plots and scratch morphologies under three typical height difference configurations. When h = 0 mm, both abrasive particles simultaneously contact the workpiece and perform cutting, resulting in a symmetrical stress field distribution. The high-stress zones beneath the left and right abrasive particles exhibit comparable intensities, forming a distinct “bipolar” stress distribution pattern. Surface micrographs reveal that both scratches share consistent depths and similar edge chipping, indicating excellent synchrony in material removal. However, stress superposition occurs in the inter-abrasive region, with stress at point M reaching 430 MPa. This indicates significant mechanical interference due to the high overlap of the two abrasives’ active zones. While this configuration ensures consistent cutting depth, the high stress concentration readily induces cumulative subsurface damage.

The primary reason for the aforementioned difference in results lies in how the height difference between abrasive grains alters the spatiotemporal sequence of material removal and the propagation path of the stress field. Under the equal-height configuration, both abrasive grains act simultaneously on the workpiece, causing their stress fields to spatially superimpose and form a continuous high-stress zone, thereby exacerbating the tendency toward brittle fracture of the material. However, introducing a height difference decomposes the cutting process into two stages: “primary cutting followed by secondary finishing.” The higher abrasive first performs deep cutting, whose generated stress field and damage zone partially “pre-treat” the material, reducing local resistance. The lower abrasive subsequently performs shallow cutting on the softened area, requiring lower cutting force and primarily removing material through plastic shearing, thereby improving surface quality.

The introduction of height differences also alters the chip formation mechanism. Under the equal-height configuration, chips are produced in a “chunky” form, indicating that material removal is dominated by brittle fracture. In contrast, under the h ≥ 0.01 mm configuration, chips exhibit “continuous ribbon-like” or “powdery” characteristics, suggesting that plastic shearing and microfracturing become the dominant mechanisms. This transition is intrinsically consistent with improved machined surface quality, providing both experimental and theoretical grounds for optimizing material removal patterns through regulation of the height difference between abrasive grains.

**Figure 11:**
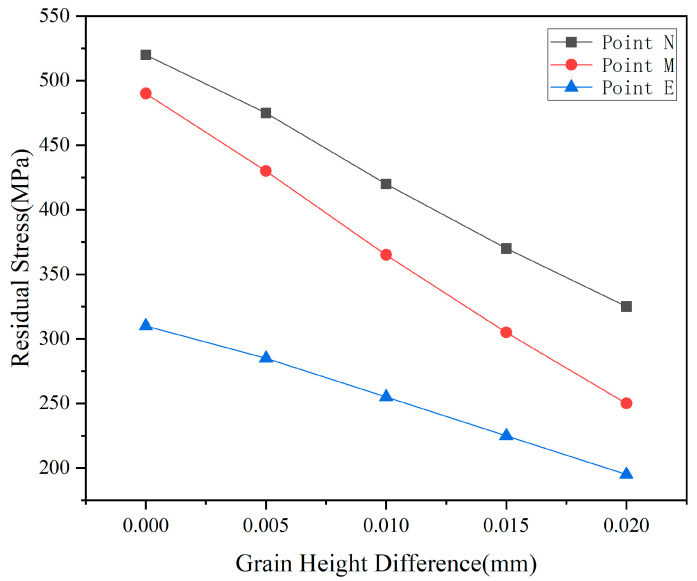
Residual stress at nodes as a function of abrasive height difference.

Figure 11 quantitatively illustrates the variation in residual stress at three monitoring points with the height difference of abrasive particles, revealing the dynamic process of stress field decoupling. Stress in the primary cutting zone at point N exhibits an initial increase followed by stabilization as the height difference increases: rising from 520 MPa to approximately 530 MPa, then stabilizing at around 325 MPa for h > 0.01 mm. This phenomenon indicates that when h < 0.01 mm, the higher-positioned abrasive grain bears a greater share of the cutting load, leading to a slight increase in stress concentration. However, once the height difference exceeds a critical value, the cutting depth of the higher-positioned grain stabilizes, and the stress level consequently stabilizes as well.

Comparing the three curves reveals a distinct “inflection point” within the range of h = 0.01–0.015 mm, indicating this interval represents a critical transition zone where the coupling effect between abrasive particles weakens. Within this range, the spatial distribution of the stress field shifts from “continuous superposition” to “discrete independence,” and the material removal mechanism transitions from “cooperative brittle fracture” to “sequential plastic flow.” This provides a crucial process parameter window for optimizing the abrasive arrangement in diamond wire saws.

From the perspective of stress control and surface quality, it is recommended to set the abrasive particle height difference within the range of 0.008–0.015 mm. This range effectively reduces coupling stress between particles—lowering stress at point M by 40–45%—while maintaining a reasonable material removal rate. It represents the optimal choice for balancing machining efficiency and surface quality. For ultra-precision machining applications, the height difference may be appropriately increased to 0.015–0.02 mm to maximize suppression of surface and subsurface damage, though this entails a corresponding reduction in material removal rate.

#### 3.2.2. Analysis of Cutting Forces at Different Height Differences

**Figure 12:**
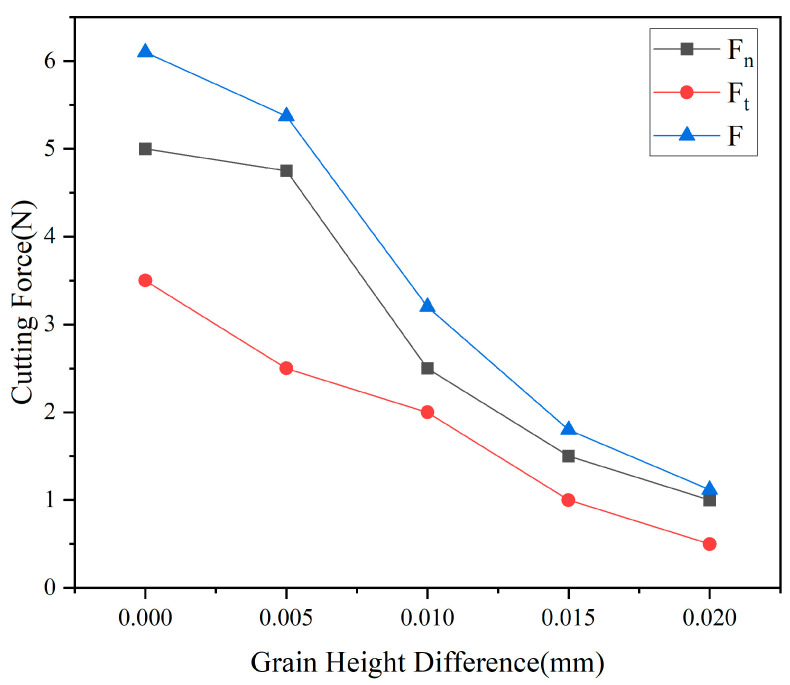
Relationship between different height differences of abrasive grains and cutting force.

Figure 12 reveals the influence pattern of particle height difference on the three cutting force components, exhibiting distinctly different mechanical response characteristics compared to radial spacing. Under the equal-height configuration h = 0, the normal force Fn is 5.0 N, the tangential force Ft is 3.5 N, and the resultant force F is 6.1 N, with the tangential force ratio Ft/Fn reaching as high as 0.70. This elevated tangential force ratio indicates that when both abrasive grains cut simultaneously, plastic shear dominates material removal. However, due to the intense stress field superposition, the high density of microcracks within the material results in suboptimal actual cutting efficiency.

The underlying mechanism of this phenomenon can be explained from the perspective of abrasive-workpiece contact mechanics and energy distribution. Under equal-height configurations, synchronous cutting by two abrasive grains results in high instantaneous cutting power. Although the tangential force dominates, significant energy is consumed by crack propagation induced by stress field superposition, limiting the actual material removal efficiency. Introducing a moderate height difference decouples the cutting process temporally. The higher-positioned abrasive grains undertake the primary material removal task, concentrating their cutting power on plastic deformation and shearing. However, an excessive height difference (h > 0.015 mm) results in an overly shallow cutting depth for the lower-positioned grains. Their cutting action is primarily confined to the surface layer within 2–5 μm. While this achieves an excellent surface finish, the material removal rate drops significantly. At this point, the effective number of abrasive grains per unit length is effectively halved, leading to a significant reduction in machining efficiency. Furthermore, as high-positioned abrasive grains bear nearly the entire cutting load, their wear rate accelerates, shortening tool life. From a long-term machining perspective, this proves economically inefficient.

From the perspective of dynamic cutting force response, the nonlinear characteristics of the force-height difference curve in Figure 12 reveal the complex dynamic interactions between abrasive particles. Within the range h < 0.01 mm, the cutting force decreases rapidly with increasing height difference, exhibiting a slope of approximately −300 N/mm. This indicates that the release of particle coupling effects during this stage significantly contributes to reducing cutting force. Within the range h > 0.01 mm, the decrease in cutting force slows, with the slope reducing to approximately −50 N/mm, indicating diminishing marginal benefits from further increasing the height difference. This inflection point at h ≈ 0.01 mm aligns with the critical height difference identified in stress field analysis, further validating the pivotal role of this parameter in process optimization.

### 3.3. Abrasive Particle Distribution Analysis

Regarding the radial spacing between abrasive particles, as the spacing increased from 0.01 mm to 0.05 mm, the residual stress at point N decreased from 485 MPa to 290 MPa, a reduction of 40%, while the stress at point M decreased from 460 MPa to 110 MPa, and at point E from 275 MPa to 135 MPa. This indicates that increasing the abrasive spacing significantly mitigates the stress field superposition effect, effectively reducing residual stress levels and damage severity in both the machined surface and subsurface layers. However, cutting forces exhibited an opposite trend: the normal cutting force increased from 13.5 N to 20.5 N, the tangential cutting force rose from 2.5 N to 6.5 N, and the total cutting force surged from 13.73 N to 21.51 N—a 57% increase. This indicates that the expanded independent action zone of abrasive grains led to increased load-bearing capacity per grain. Balancing the dual objectives of stress control and cutting force optimization, a recommended radial spacing range of 0.02–0.03 mm achieves significant stress reduction with relatively moderate cutting force increase, realizing a process equilibrium of “low damage–controllable load.”

Regarding the height differences of abrasive particles, as the height difference increased from 0 mm to 0.02 mm, the stress at point N decreased from 520 MPa to 325 MPa, a reduction of 37%. The stress at point M decreased from 490 MPa to 250 MPa, and the stress at point E decreased from 310 MPa to 195 MPa. This confirms that the vertical misalignment of abrasive particles effectively decouples the stress superposition mechanism. More significantly, cutting forces exhibited a monotonically substantial decrease: normal force dropped from 5.0 N to 1.0 N, tangential force decreased from 3.5 N to 0.5 N, and resultant force fell from 6.10 N to 1.12 N—an 82% reduction. This demonstrates the height difference parameter’s optimization potential for multi-abrasive synergistic cutting processes. This approach reduces instantaneous loads while improving surface integrity. Based on the dual optimization effects of stress and cutting force, a height difference range of 0.01–0.02 mm is recommended. Under these conditions, significant benefits of over 30% reduction in residual stress and over 75% reduction in cutting force can be achieved, providing clear parameter guidance for enhancing monocrystalline silicon cutting quality and efficiency.

During actual cutting processes, the wear evolution of diamond abrasive grains significantly influences material removal mechanisms and stress field distribution characteristics. The finite element model developed in this study, based on the rigid abrasive particle assumption, does not account for the dynamic effects of wear, which, to some extent, affects the absolute accuracy of the simulation results. The wear evolution of abrasive particles primarily involves the microfracturing and blunting of sharp cutting edges, leading to the formation of a rounded radius at the particle tip. Consequently, the cutting mode gradually transitions from shear removal to extrusion plowing. This transition alters the stress field distribution pattern: sharp abrasive particles generate “peak-type” stress concentrations beneath the cutting edge, while worn and blunted particles exhibit increased contact area, shifting the stress distribution to a “plateau-type” pattern. This reduces peak stress but increases the depth of influence. According to indentation fracture mechanics theory, the increased blunting radius due to wear raises the critical cutting depth, favoring ductile material removal. Furthermore, the non-uniformity in abrasive wear rates causes the initially uniform abrasive layout to naturally evolve into minute height differences during cutting. This aligns with the stress field evolution patterns shown in Figure 6 and Figure 10, where the introduction of height differences objectively mitigates the stress field superposition effect.

In actual diamond wire saw cutting processes, an abrasive layout scheme combining radial spacing d = 0.02–0.03 mm with height difference h = 0.01–0.02 mm should be adopted. This parameter combination achieves synergistic optimization across multiple objectives—stress control, cutting force efficiency, and processing productivity. It significantly suppresses subsurface damage depth, reduces microcrack density, and extends abrasive grain lifespan, thereby establishing a foundational process for high-quality fabrication of photovoltaic and semiconductor-grade monocrystalline silicon wafers.

## 4. Optimization of Multi-Particle Cutting Parameters

Diamond wire saw cutting, as a core technology in monocrystalline silicon wafer processing, directly impacts the subsequent application performance of the wafers. During the cutting process, factors such as the geometric parameters of the abrasive grains and process parameters significantly influence cutting forces and surface/subsurface damage. Excessive cutting forces can cause severe subsurface damage, increasing material removal during subsequent grinding and polishing. Meanwhile, residual stresses affect wafer flatness and service life. Therefore, investigating the influence of cutting parameters on cutting forces and residual stresses is crucial for optimizing diamond wire saw cutting processes [33]. This chapter employs finite element simulation software to analyze, through orthogonal experimental design, how process parameters—including cutting speed, abrasive cone angle, and penetration depth—affect cutting forces and residual stresses during multi-abrasive diamond wire cutting. Furthermore, the Non-dominated Sorting Genetic Algorithm III (NSGA-III) genetic algorithm is utilized to identify the equilibrium relationship between cutting forces and residual stresses, thereby deriving an optimized parameter scheme featuring low cutting forces and low residual stresses.

### 4.1. Orthogonal Experimental Design for Multi-Particle Cutting

This section employs a dual-abrasive layout scheme with radial spacing d = 0.02 mm and height difference h = 0.01 mm. Simulation experiments were conducted using an L16 orthogonal array, with values assigned to three factors as shown in Table 3. The controlled variables are linear speed U, abrasive cone angle V, and diamond particle penetration depth W. Through finite element analysis and orthogonal testing, 16 data samples were collected. Consequently, the simulation results for silicon monocrystal cutting, featuring two response characteristics—residual stress and cutting force—are presented in Table 4.

### 4.2. Effect of Cutting Parameters on Residual Stresses in Monocrystalline Silicon

Table 5 presents the primary factors influencing residual stresses on single-crystal silicon surfaces. As shown in Table 5, the indentation depth W of diamond particles, the abrasive cone angle V, and the linear speed U exert a significant influence on the residual stress of the product. The magnitude of their effects on residual stress is determined by the range values: W > U > V. This indicates that indentation depth is the most critical factor affecting residual stress, followed by linear speed, while the abrasive cone angle has a relatively minor impact.

**Figure 13:**
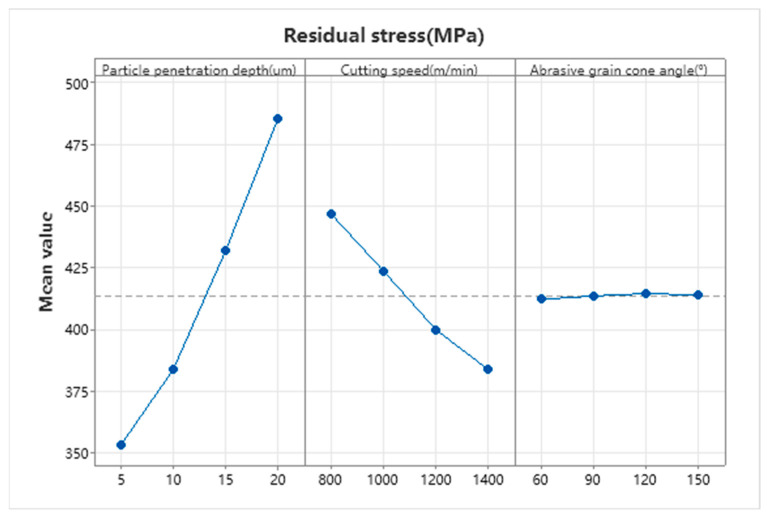
Residual stress principal effect diagram.

The main effects plot in Figure 13 reveals the mechanisms of action for each parameter. The indentation depth W of diamond particles exhibits a significant positive correlation: as the indentation depth increases from 5 μm to 20 μm, the residual stress rises sharply from 352.8 MPa to 485.8 MPa, representing a 37.7% increase. The indentation depth directly determines the volume of the plastic deformation zone and the degree of stress concentration. According to Hertzian contact theory, an expanded contact area increases the maximum contact stress. Deeper indentation leads to more extensive lattice distortion and accumulation of dislocation density, forming a residual compressive stress field after unloading. When exceeding the brittle-ductile transition critical depth, material removal shifts from ductile flow to brittle crack propagation, further intensifying stress concentration.

Linear velocity U exhibits a negative correlation with residual stress: increasing from 800 m/min to 1400 m/min reduces residual stress from 447 MPa to 383.7 MPa, a decrease of 14.2%. Under high strain rates, plastic deformation time shortens, microcrack propagation is suppressed, and the formation of macroscopic crack networks diminishes. Simultaneously, frictional heat and plastic work elevated cutting zone temperatures to 300–500 °C, lowering dislocation slip activation energy and promoting stress release through thermal softening. The abrasive cone angle V exerted minimal influence on residual stress: within the 60–150° range, residual stress fluctuated between 412.3 and 414.6 MPa, with a mere 2.3 MPa variation. At the smaller 60° cone angle, extrusion-plowing dominates, causing severe plastic deformation but limiting crack propagation. At 150°, the shear component increases, reducing the stress concentration zone. The competition between these effects renders residual stresses insensitive to cone angle.

### 4.3. Effect of Cutting Parameters on Cutting Forces in Monocrystalline Silicon Machining

Table 6 presents the mean response table for cutting forces in monocrystalline silicon machining. It reveals that the indentation depth of diamond particles, the abrasive cone angle, and the linear speed exert a profound influence on cutting forces. The impact of these three cutting parameters on cutting forces follows the order: W > U > V, consistent with the influence ranking of residual stresses. However, their underlying mechanisms differ significantly.

**Figure 14:**
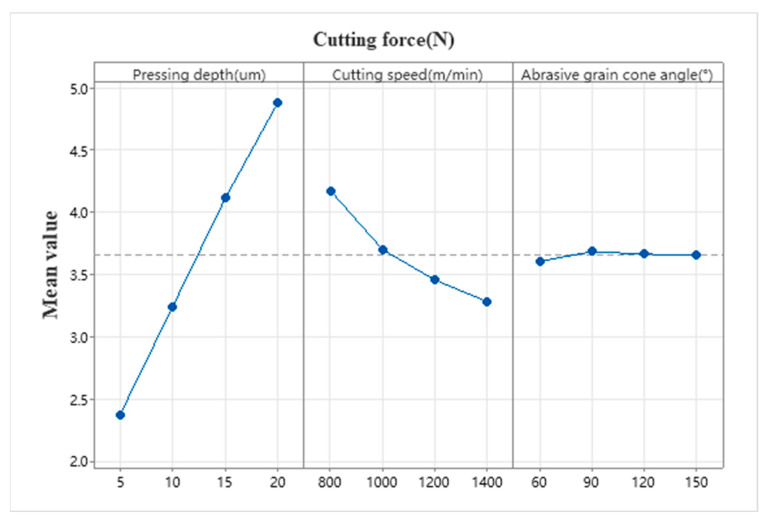
Principal effect plot of cutting forces.

As shown in the main effects plot in Figure 14, the penetration depth W of diamond particles exhibits the most significant and strongly positive correlation with cutting force: when the penetration depth increases from 5 μm to 20 μm, the cutting force surges dramatically from 2.38 N to 4.88 N, representing a 105% increase. This trend aligns with classical cutting force theory, where increased penetration depth directly expands the cross-sectional area of material removal. Greater penetration depth drives abrasive cutting into the “fully plastic regime,” where material removal primarily involves continuous shearing and large-scale plastic flow, substantially increasing the required shear work and friction work. Finite element simulations reveal that as the penetration depth increases from 5 μm to 20 μm, the ratio of normal force to tangential force rises from 1.2 to 1.5. This indicates a significant strengthening of the extrusion-plowing mechanism, with pronounced built-up edges and plastic ridges forming ahead of the abrasive grains, leading to a reduction in the effective rake angle.

Cutting speed U exhibits a negative correlation with cutting force: when increased from 800 m/min to 1400 m/min, cutting force decreased from 4.18 N to 3.28 N, representing a 21.5% reduction. This phenomenon can be explained through three mechanisms: First, the strain rate softening effect: high-speed cutting shortens the material removal time scale, inhibiting microcrack propagation and inducing a relatively “ductile” removal mode. Second, the thermal softening mechanism: as the cutting temperature rises from 150 °C to 350 °C, the critical cutting stress of monocrystalline silicon decreases by approximately 15–20%, reducing the activation energy for dislocation slip. Third, the altered contact state between abrasive grains and chips: accelerated chip flow velocity shortens the contact length on the rake face, reducing friction forces. The influence of abrasive grain cone angle V on cutting force is extremely negligible: within the 60–150° range, cutting force remains virtually unchanged between 3.61 N and 3.69 N, with a mere variation of 0.08 N. This occurs because actual abrasive cutting edges possess micrometer-level blunting radii. When the penetration depth matches the blunting radius magnitude, the nominal cone angle’s effect is masked by the blunting phenomenon, with cutting forces primarily determined by edge extrusion and plowing. Additionally, while a small cone angle increases the squeezing force, it also expands the stress dispersion zone. Conversely, a large cone angle reduces the squeezing force but concentrates stress more intensely at the cutting-edge’s tip. These two effects counteract each other.

Cutting power is primarily consumed in three components: plastic deformation work, friction work, surface energy, and crack propagation energy. High-speed cutting increases the proportion of plastic deformation work to 65–70% while reducing friction work to 20–25%, reflecting a fundamental shift in the material removal mechanism. Cutting forces exhibit dynamic fluctuations: during the initial penetration phase, cutting forces rise rapidly with a slope of approximately 20–30 N/ms; in the steady-state cutting phase, forces remain near the average value with periodic fluctuations of 10–20%, caused by dislocation avalanches and intermittent microcrack propagation; during the unloading phase, cutting forces rapidly decrease to zero. Fluctuations with ΔF < 15% correspond to plastic flow, yielding good surface quality; ΔF > 30% indicates brittle fracture. Based on Merchant’s theory, the cutting force can be expressed as: Fc = τ_s_ × A_shear_/sin(φ) + σ_y_ × A_plow_ + μ × Fn, where the shear term represents effective removal resistance, the plowing term relates to blunting, and the friction term can be reduced through air film formation.

### 4.4. NSGA-III Multi-Objective Optimization

#### 4.4.1. Regression Model

In diamond wire saw cutting, the target response function exhibits significant variations with changes in multiple control factors, rather than a simple linear relationship, making its analytical model difficult to obtain. To address this issue, one approach involves incorporating control factors into the response statistics based on regression analysis. The resulting model can be expressed by Equation (10):

(10) $$y\left(x\right)=c_{0}+\sum_{i=1}^{n}c_{i}x_{i}+\sum_{ij(i<j)}^{n}c_{ij}x_{i}x_{j}$$

In the equation [34], *y* denotes the response function; $c_{0}$, $c_{i}$, and $c_{ij}$ represent the zero-order, first-order, and second-order coefficients, respectively; and $x_{i}$ and $x_{j}$ denote the control variables, with n being the number of factors.

A regression model for monocrystalline silicon cutting was designed using the commercial data analysis software Minitab 22. The regression models for residual stress R_s_ and cutting force F_c_ are expressed as Equations (11) and (12):

R_s_ = −852.3 + 0.425 U − 1.82 V + 35.6 W − 0.00125 U^2^ + 0.0068 V^2^ − 0.52 W^2^ + 0.00085 UV − 0.0165 UW + 0.105 VW (11)

F_c_ = −5.68 + 0.0052 U − 0.025 V + 0.485 W − 0.0000018 U^2^ + 0.000065 V^2^ − 0.0085 W^2^ + 0.000012 UV − 0.000095 UW + 0.0018 VW (12)

#### 4.4.2. Two-Objective Optimization by NSGA-III

NSGA-III is an improved algorithm based on NSGA-II. It maintains convergence and diversity through non-dominated sorting and reference point elite strategies, featuring adaptive capabilities for dynamically removing and incorporating new reference points. This study employs NSGA-III to optimize the dual-objective problem of residual stress and cutting force in diamond wire sawing [35]. Generally, residual stress and cutting force exhibit inherent trade-offs. Therefore, this research adopts a combined model of residual stress and cutting force as the dual-objective optimization framework. The operational parameters are set as follows:

(1) fmin = {Rs, Fc}
(2) Population = 100
(3) Maximum number of iterations = 500
(4) Cross-probability = 0.5
(5) Probability of mutation = 0.0005

Table 7 presents selected values from the dual-objective optimization Pareto frontier solutions. As shown in Table 7, different parameter combinations achieve distinct equilibrium states between residual stress and cutting force. For instance, Scheme NO.1 employs a high linear speed of 1385 m/min, a large cone angle of 142°, and a small penetration depth of 6.2 μm, achieving a low residual stress of 332.5 MPa and a cutting force of 2.15 N. In contrast, Scheme NO.5 utilizes a moderate parameter combination, attaining a favorable balance between residual stress and cutting force.

### 4.5. Model Validation

**Figure 15:**
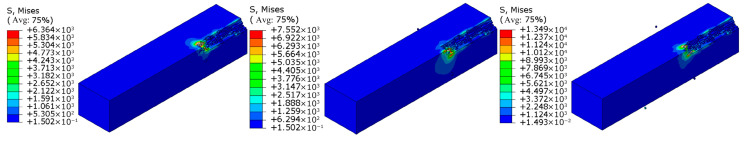
Schematic diagram of selected simulation results.

**Figure 16:**
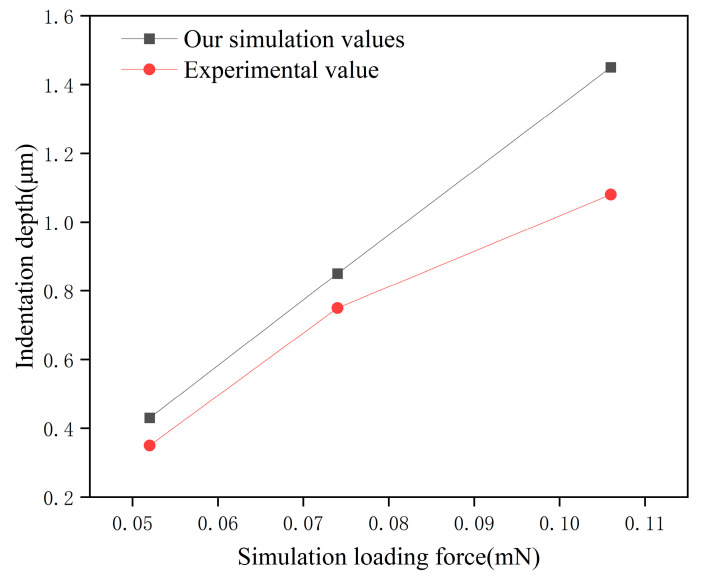
Comparison of simulation results with experimental data.

To validate the reliability of the simulation model, this study conducted simulation analysis under experimental conditions referenced from [36]. As shown in Figure 15, the experimental parameters in this literature are similar to those in this study, providing good comparability. Comparison of the simulation results with the literature experimental data revealed a high degree of agreement, thereby validating the reliability of the model developed in this paper. Our simulation results are compared with their experimental results, as shown in Figure 16.

Figure 16 shows the comparison between simulated depth values and experimental depth values under identical contact stress in Hertzian units. The simulated results align with the experimental trends, which verifies the reliability of the simulation model.

## 5. Conclusions

This study established a three-dimensional finite element model for diamond multi-abrasive multi-scratch machining of monocrystalline silicon. It systematically revealed the coupled mechanism of residual stress and cutting force influenced by abrasive layout and cutting parameters, and obtained a process coordination scheme through multi-objective optimization. The main conclusions are as follows:

(1) When the radial clearance increases from 0.01 mm to 0.05 mm, the stress at the intermediate monitoring point M decreases significantly from 460 MPa to 110 MPa. However, the combined cutting force increases from 13.73 N to 21.51 N. The optimized recommendation for the radial clearance d is 0.02–0.03 mm, achieving a “low damage–controllable load” balance characterized by low residual stress and reduced cutting force.
(2) Introducing a height difference of h = 0.01–0.02 mm transforms the cutting mode from “synchronous brittle fracture” to “time-division plastic trimming,” resulting in a significant reduction in stress intensity. The normal force and tangential force decrease by 80% and 86%, respectively, enabling a reduction in residual stress exceeding 30%.
(3) The penetration depth W is the absolute primary factor: increasing from 5 μm to 20 μm resulted in residual stress and cutting force increases of 37.7% and 105%, respectively. The linear speed U exhibits a negative correlation effect: increasing from 800 m/min to 1400 m/min caused residual stress and cutting force to decrease by 14.2% and 21.5%, respectively, due to strain rate softening and thermal softening mechanisms. The abrasive grain cone angle V has a negligible influence, as the blunting effect of the cutting edge masks the geometric cone angle’s impact.
(4) The Pareto frontier obtained via the NSGA-III algorithm reveals a positive correlation between residual stress and cutting force. The optimal solution with U = 1385 m/min, V = 142°, and W = 6.2 μm achieves dual low targets of 332.5 MPa residual stress and 2.15 N cutting force. Engineering applications are recommended to adopt an abrasive layout with d = 0.02–0.03 mm and h = 0.01–0.02 mm, combined with parameters U = 1100–1250 m/min and W = 10–15 μm. This configuration simultaneously satisfies multiple objectives: low residual stress, minimal cutting force, and high abrasive effectiveness.

## Figures and Tables

**Table 1:** Physical properties of monocrystalline silicon and diamond.

Properties	Monocrystalline Silicon	Diamond
Density (T/mm^3^)	2.339·10^−9^	2.515·10^−9^
Modulus of elasticity (MPa)	1.9·10^5^	1.03·10^6^
Poisson ratio	0.28	0.06
Thermal conductivity (T/(mm·s^3^·K))	150	1300
Thermal expansion (°C^−1^)	2.6·10^−6^	2.2·10^−6^
Specific heat capacity (mJ/T/°C)	7·10^8^	9.2·10^5^

**Table 2:** Constitutive parameters of monocrystalline silicon JH-II.

Parameters	Value	Parameters	Value
ρ (T/mm^3^)	2.339·10^−9^	A	0.95
Modulus of elasticity (MPa)	1.9·10^5^	B	0.35
D_1_	0.35	C	0.009
D_2_	0.74	M	1.0
FS	2.0	N	0.67
K1 (MPa)	220,000	EPSI	1.0
K2 (MPa)	360,000	T (MPa)	700
K3 (MPa)	0	SF_MAX_/MPa	800
ε˙_0_	1.0	HEL (MPa)	15,000
		P_HEL_ (MPa)	6000

**Table 3:** Control factors and level standard setting.

Level	Cutting Speed (m/min)	Abrasive Grain Cone Angle (°)	Diamond Particle Indentation Depth (μm)
	U	V	W
1	800	60	5
2	1000	90	10
3	1200	120	15
4	1400	150	20

**Table 4:** Experimental design and response statistics table.

No.	Cutting Speed (m/min)	Abrasive Grain Cone Angle (°)	Diamond Particle Indentation Depth (μm)	Residual Stress (MPa)	Cutting Force (N)
1	800	60	5	385.2	2.85
2	800	90	10	421.6	3.92
3	800	120	15	468.5	4.65
4	800	150	20	512.8	5.28
5	1000	60	10	392.7	3.15
6	1000	90	5	358.4	2.42
7	1000	120	20	495.3	4.98
8	1000	150	15	448.9	4.25
9	1200	60	15	412.5	3.88
10	1200	90	20	476.2	4.72
11	1200	120	5	342.6	2.18
12	1200	150	10	368.5	3.05
13	1400	60	20	458.7	4.55
14	1400	90	15	398.3	3.68
15	1400	120	10	352.1	2.85
16	1400	150	5	325.8	2.05

**Table 5:** Residual stress mean value response table.

Level	U	V	W
1	447	412.3	352.8
2	423.8	413.6	383.7
3	400	414.6	432.1
4	383.7	414.0	485.8
Delta	63.3	2.3	133
Ranking	2	3	1

**Table 6:** Average cutting force response table.

Level	U	V	W
1	4.18	3.61	2.38
2	3.7	3.69	3.24
3	3.46	3.66	4.12
4	3.28	3.66	4.88
Delta	0.9	0.08	2.5
Ranking	2	3	1

**Table 7:** Partial two-objective optimization Pareto frontier solutions.

No.	U (m/min)	V (°)	W (μm)	R_s_ (MPa)	F_c_ (N)
1	1385	142	6.2	332.5	2.15
2	1320	135	7.8	348.7	2.58
3	1245	128	9.5	365.2	2.95
4	1180	118	11.2	382.6	3.35
5	1105	108	12.8	391.5	3.72
6	1025	95	14.5	425.8	4.18
7	950	82	16.3	448.3	4.65

## Data Availability

The raw data supporting the conclusions of this article will be made available by the authors on request.
